# The role of historical and contemporary processes on phylogeographic structure and genetic diversity in the Northern Cardinal, *Cardinalis cardinalis*

**DOI:** 10.1186/1471-2148-11-136

**Published:** 2011-05-20

**Authors:** Brian Tilston Smith, Patricia Escalante, Blanca E Hernández Baños, Adolfo G Navarro-Sigüenza, Sievert Rohwer, John Klicka

**Affiliations:** 1 School of Life Sciences, University of Nevada, Las Vegas, 4505 S. Maryland Parkway Box 454004 Las Vegas, NV 89154, USA; 2 Marjorie Barrick Museum of Natural History, University of Nevada, Las Vegas, 4505 S. Maryland Parkway Box 454012, Las Vegas, NV 89154-4012, USA; 3 Instituto de Biología, Universidad Nacional Autónoma de México, México, D.F., México; 4 Museo de Zoología, Facultad de Ciencias, Universidad Nacional Autónoma de México, Apartado Postal 70-399, México DF 04510, México; 5 Burke Museum and Department of Biology, University of Washington, Seattle, Washington, USA

## Abstract

**Background:**

Earth history events such as climate change are believed to have played a major role in shaping patterns of genetic structure and diversity in species. However, there is a lag between the time of historical events and the collection of present-day samples that are used to infer contemporary population structure. During this lag phase contemporary processes such as dispersal or non-random mating can erase or reinforce population differences generated by historical events. In this study we evaluate the role of both historical and contemporary processes on the phylogeography of a widespread North American songbird, the Northern Cardinal, *Cardinalis cardinalis*.

**Results:**

Phylogenetic analysis revealed deep mtDNA structure with six lineages across the species' range. Ecological niche models supported the same geographic breaks revealed by the mtDNA. A paleoecological niche model for the Last Glacial Maximum indicated that cardinals underwent a dramatic range reduction in eastern North America, whereas their ranges were more stable in México. In eastern North America cardinals expanded out of glacial refugia, but we found no signature of decreased genetic diversity in areas colonized after the Last Glacial Maximum. Present-day demographic data suggested that population growth across the expansion cline is positively correlated with latitude. We propose that there was no loss of genetic diversity in areas colonized after the Last Glacial Maximum because recent high-levels of gene flow across the region have homogenized genetic diversity in eastern North America.

**Conclusion:**

We show that both deep historical events as well as demographic processes that occurred following these events are critical in shaping genetic pattern and diversity in *C. cardinalis*. The general implication of our results is that patterns of genetic diversity are best understood when information on species history, ecology, and demography are considered simultaneously.

## Background

A multitude of studies of various North American taxa have shown that historical events, such as Pleistocene climatic changes coupled with topographic, hydrologic and ecological barriers, were instrumental in generating phylogeographic structure within species [1-4]. From the extensive body of phylogeographic work on North American species, two generalizations have emerged 1) taxa were isolated into independently evolving lineages (i.e. species, subspecies, phylogroups) during the Pleistocene [5] and 2) genetic diversity within species is highest in areas that remained stable (refugia) through glacial cycles [6]. Ultimately, these hypothesized predictions seek to link the phylogeographic structure in species to historical events.

A recent and critical historical event was the Last Glacial Maximum, a time when ice sheets covered much of present-day temperate North America. Entire biotas were fragmented and displaced to more southern latitudes [6]. After the glaciers receded certain species colonized these newly available areas by rapid long-distance dispersal, a phenomenon referred to as the leading edge or pioneer model [7]. A major prediction of the leading edge model is that genetic diversity will be lower in the recently colonized areas. This idea is supported by the observation that many species have higher genetic diversity in European refugial areas [6]. Additionally, genetic data indicate that historical population expansions occurred in many species, a finding consistent with species colonizing newly available areas with suitable habitat. A goal of phylogeography is to recover evidence of population expansions; however, the lag between earlier population expansions and the present represents a time when ongoing demographic processes can either erase earlier genetic signatures of population structure or expansions or prevent such signatures from developing at all.

Contemporary processes can affect the spatial distribution of genetic diversity in various ways. High levels of gene flow may homogenize haplotype diversity throughout a population that once showed genetic structure. Conversely, nonrandom mating with individuals in close geographic proximity can generate genetic structuring within a continuous population [8]. Autecology also plays a critical role in how species maintain genetic connectivity across the landscape [9,10]. Vagile species with high ecological thresholds will be able spread haplotypes across an area more readily than poor dispersers with narrow ecological requirements. Interpretations of genetic diversity patterns are further confounded by changes in gene flow due to oscillations in population densities and by shifts in the connectivity of habitats. These points highlight the complexity of population-level processes and emphasize that genetic patterns of diversity may become so tightly coupled to on-going processes that signatures of historical differences or similarities may be erased.

To examine the impact of historical and contemporary processes on genetic pattern and diversity, we investigated a broadly distributed North American songbird, the Northern Cardinal (*Cardinalis cardinalis*). Cardinals are distributed from southeastern Canada, throughout the eastern United States, and in the southwestern states south through the Mexican lowlands to northern Central America [11]. *Cardinalis cardinalis *is currently divided into 18 subspecies representing four subspecies groups [11]. The species inhabits multiple biomes which include deserts, dry and humid tropical forests, and deciduous temperate forests. In all regions it prefers similar habitats of shrubs, small trees, forest-edge, or secondary growth [11].

In this study we use mitochondrial DNA (mtDNA) sequences, ecological niche models, and demographic data to test the impacts of historical and contemporary processes on pattern of present-day genetic differentiation and diversity in *C. cardinalis*. First, we evaluated whether historical events had generated deep phylogeographic structure or monophyletic groups in mtDNA across the range of cardinals. Second, we tested for the signature of historical demographic expansions that should have been generated in populations that expanded following glacial retreat. Third, we used present-day and paleoecological niche models to explore if clades had geographically stable ranges since the Last Glacial Maximum. Finally, we evaluate if genetic diversity patterns can be explained by contemporary demographic processes.

## Results

### Genetic Diversity and Pattern

**Figure 1:**
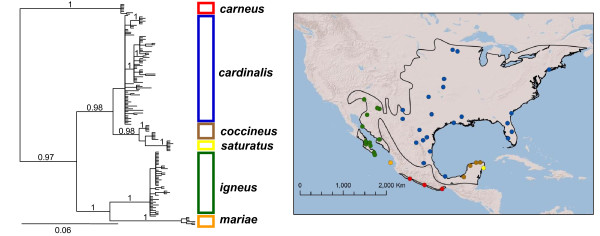
**Bayesian mitochondrial gene tree for *Cardinalis cardinalis *with posterior probabilities**. Sampled localities for each clade are plotted on the accompanying map of North America. *carneus *(red); c*ardinalis *(blue); *coccineus *(brown); *saturatus *(yellow); *igneus *(green); *mariae *(orange).

The best-fit models of sequence evolution were GTR + I + G for the complete ND2 gene, GTR + I for the 1^st ^and 2^nd ^codons and GTR + I +G for the 3^rd ^codon. Both the partitioned and unpartitioned analyses yielded trees with similar topologies but with different likelihoods. The partitioned dataset (lnL -5082.52) was a better fit to the data than the unpartitioned data (lnL -5354.79) with a ln Bayes factor of 272.27. Although the harmonic mean method can be sensitive to a high degree of variance [12-14], the Bayes factors reported here are informative because the likelihoods of the two mtDNA gene trees are well separated [15]. The mtDNA tree strongly indicated deep genetic structure across the range of *C. cardinalis *with no mtDNA haplotype sharing among regions except potentially in the Mexican Gulf coast region (Figure 1). Most major nodes in the tree had posterior probabilities greater than 0.95. Exceptions were the node for the *cardinalis *clade (pp < 0.50) and the lack of reciprocal monophyly between the *coccineus *and *saturatus *clades. Uncorrected node to internode genetic distance for each clade ranged from 0.35 - 2.60% (Table 1).

**Table 1:** Clade genetic and range characteristics

Claudea	Range Size (sq km)	n	Hap	Hap Diversity	Nuc Diversity	Nuc Diversity*	Genetic Dist
*cardinalis*	3,189,130	82	48	0.9530	0.00434	0.00397	0.75
*carneus*	35,165	8	3	0.4643	0.00065	0.00083	2.60
*coccineus*	204,229	11	3	0.6364	0.00175	0.00186	0.35
*igneus*	444,297	47	21	0.8608	0.00197	0.00179	1.65
*mariae*	245	6	2	0.7333	0.00173	0.00173	1.65
*saturatus*	647	8	2	0.2500	0.00024	0.00032	0.35

Summary of genetic diversity indices and range sizes for the six *Cardinalis cardinalis *clades. Number of individuals (n). Number of unique haplotypes (Hap). Metric on the proportion of unique haplotypes in a clade (Hap diversity). Nucleotide diversity with complete clade sampling (Nuc Diversity). Nucleotide diversity with equal sampling per clade (Nuc Diversity*). Node to internode genetic distance based uncorrected p-distances (Genetic Dist).

**Figure 2:**
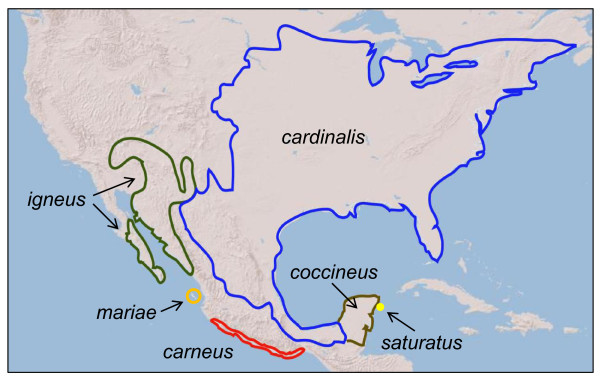
**Approximated range limits of the six *Cardinalis cardinalis *clades based on mtDNA**. *carneus *(red); c*ardinalis *(blue); *coccineus *(brown); *igneus *(green); *saturatus *(yellow); *mariae *(orange).

The genetic breaks are largely concordant with the four long-recognized morphological groups: *carneus*, *igneus*, *coccineus*, and c*ardinalis *[11] with the addition of two monophyletic island lineages, *saturatus *within c*occineus *and m*ariae *within *igneus *(Figure 1, Figure 2). Molecular dating from mtDNA indicated that *C. cardinalis *diverged from the Miocene to Late Pleistocene. The basal split in the mtDNA tree separates the *carneus *clade from all other cardinals occurred 3.41 million years ago (95% HPD: 0.99-6.78). The remaining cardinals split into western and eastern clades approximately 2.39 million years ago (95% HPD: 0.66-4.87). The western clade is comprised of a monophyletic *igneus*, distributed throughout the Baja California Peninsula, Sonoran and southern Mojave deserts and a monophyletic *mariae *on the Tres Marías Islands. *Mariae *diverged from the mainland 1.42 million years ago (95% HPD: 0.31-2.86). The eastern clade is comprised of three groups (Figure 1). *Coccineus *occurs on the Yucatán Peninsula and is paraphyletic with respect to the monophyletic *saturatus *group that occurs only on Cozumel Island. Haplotypes in northeastern México belong to *cardinalis*, although traditional taxonomy had placed specimens from this region in *coccineus*. The *cardinalis *clade is geographically widespread, distributed throughout eastern North America and potentially as far south as Veracruz, México. The first divergence in the eastern clade occurred approximately 1.2 million years ago (95% HPD: 0.27-0.27) and the second, the divergence of the Cozumel Island cardinals occurred 0.46 million years ago (95% HPD: 0.07-1.06)

Population genetic analyses revealed differing levels of genetic structure for the two more widespread clades, *igneus *of northwest México and the southwestern US and *cardinalis *of eastern North America. In *igneus *20.3% of the genetic variation was among and 79.7% was within populations (AMOVA, Table 2). There is very little structure in *cardinalis *with 97.8% of variation within and 2.2% among populations (Table 2). Nucleotide diversity within clades ranged from 0.00024 - 0.00434 (complete clade sampling) and 0.00032 - 0.00397 (equal sampling; Table 1). Nucleotide diversity within the *cardinalis *clade ranged from 0.00278 - 0.00511 and haplotype diversity was high across all geographic localities 0.900 - 1.000 (Table 3). The *igneus *clade had lower nucleotide diversity (0.00017 - 0.00368) with the lowest diversity in northern part of the range (Arizona and New México). The pattern of haplotype distributions was low haplotype diversity (0.182) and no private haplotypes in the north (Table 3).

**Table 2:** AMOVA summary for the *cardinalis *and *igneus *clades

Source of Variation	df	ss	Variance of Components	% of Variation
*cardinalis*				
Among Populations	8	4.537	0.011	2.19
Within Populations	68	32.372	0.476	97.81
Total	76	36.909	0.487	
Fixation Index	FST:	0.022	p-value	0.028

*igneus*				
Among Populations	3	4.198	0.093	20.26
Within Populations	44	16.031	0.364	79.74
Total	47	20.229	0.457	
Fixation Index	FST:	0.203	p-value	<0.000

**Table 3:** Within clade genetic diversity

Locality	# of Ind	# of Hap	Nuc Div	Hap Div	Priv Hap
***cardinalis***					
Coahuila	8	6	0.00278	0.929	0.50
Florida/Georgia	9	7	0.00406	0.944	0.56
Kansas	5	4	0.00462	0.900	0.40
Louisiana	9	8	0.00443	0.972	0.67
Minnesota/Wisconsin	9	7	0.00406	0.917	0.56
New York	9	8	0.00422	0.972	0.67
Oklahoma	10	7	0.00312	0.933	0.40
Tamaulipas/Nuevo Leon	8	8	0.00511	1.000	0.88
Texas/New Mexico	12	10	0.00371	0.955	0.40
					
***igneus***					
Arizona/New Mexico	11	2	0.00017	0.182	0.00
Baja California Sur	13	9	0.00177	0.872	0.56
Sinaloa	19	13	0.00211	0.906	0.69
Tiburón Island	4	3	0.00368	0.833	0.67

Summary of genetic diversity within the *cardinalis *and *igneus *clades. Number of individuals (# of Ind). Number of haplotypes (# of Hap). Nucleotide diversity (Nuc Div). Haplotype diversity (Hap Div). Frequency of private haplotypes (Priv Hap).

**Figure 3:**
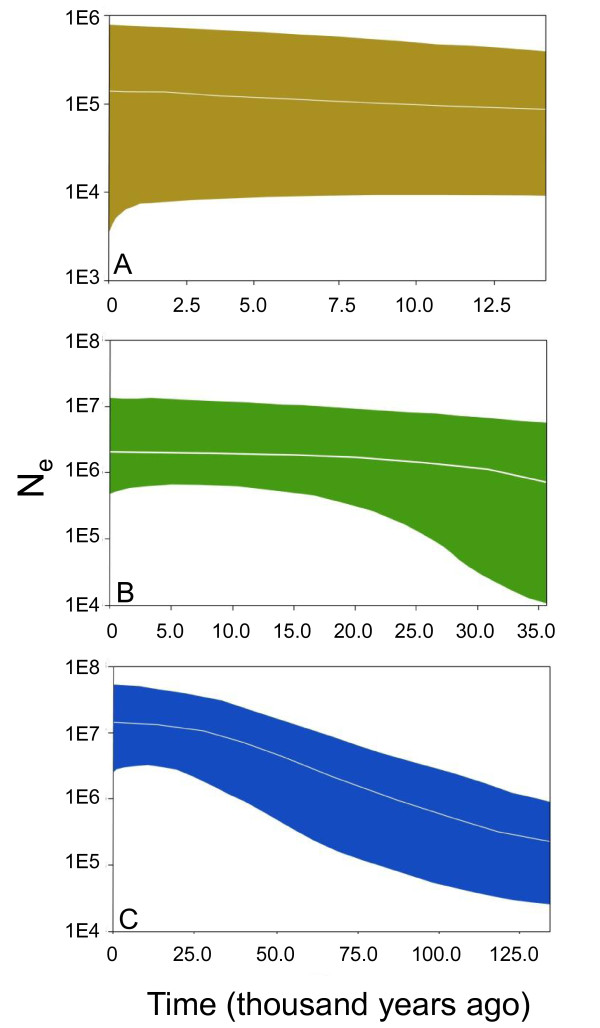
**Bayesian Skyline Plots showing change in N_e _(effective population size) across time in thousands of years**. A) *coccineus *(brown); B) *igneus *(green); C) *cardinalis *(blue).

For the historical demographic tests, the best-fit substitution model was the same for all three clades (*cardinalis*, *igneus*, and *coccineus*); HKY for 1^st ^and 2^nd ^codon positions and GTR for 3^rd ^codon position. Because *igneus *shows geographic structure (Table 2), the analysis was run without the Tiburon Island samples (*igneus *A, Table 4) and a second time without both the Tiburón Island and Baja California samples (*igneus *B, Table 4). Historical demographic tests for *igneus *(A and B) and *coccineus *did not yield robust results because the likelihood values between the constant and expansion growth models were not well-differentiated (Table 4). Lack of evidence for population expansion was further seen in Bayesian skyline plots for *igneus *and *coccineus *(Figure 3A and 3B), which were flat across time. In contrast, the Bayesian skyline plot for the *cardinalis *clade indicated population expansion over time (Figure 3C). The Bayes factors favored the expansion growth model for the *cardinalis *clade, but given the problems with estimating Bayes factors from the harmonic mean [12-14], the result must be interpreted with caution.

**Table 4:** Hypothesis testing of historical demography for Northern Cardinal clades using different demographic models

Model	Marginal Likelihood (S.E.)	log Bayes Factors
***cardinalis***		
Constant Size	-2187.800 (+/- 0.336)	-
Expansion Growth	-2170.486 (+/- 0.354)	7.520
Bayesian Skyline	-2155.803 (+/- 0.411)	13.896

***igneus *A**		
Constant Size	-1585.983 (+/- 0.316)	-
Expansion Growth	-1580.354 (+/- 0.350)	2.445
Bayesian Skyline	-1565.140 (+/- 0.364)	9.052

***igneus *B**		
Constant Size	-1513.415 (+/- 0.311)	-
Expansion Growth	-1512.251 (+/- 0.254)	0.505
Bayesian Skyline	-1503.067 (+/- 0.303)	4.494

***coccineus***		
Constant Size	-1380.049 (+/- 0.174)	-
Expansion growth	-1380.054 (+/- 0.166)	-0.002
Bayesian Skyline	-1379.877 (+/- 0.170)	0.075

*igneus *A - Tiburón Island samples were removed.

*igneus *B - Baja California and Tiburón Island samples removed

### Ecological Niche Models

**Figure 4:**
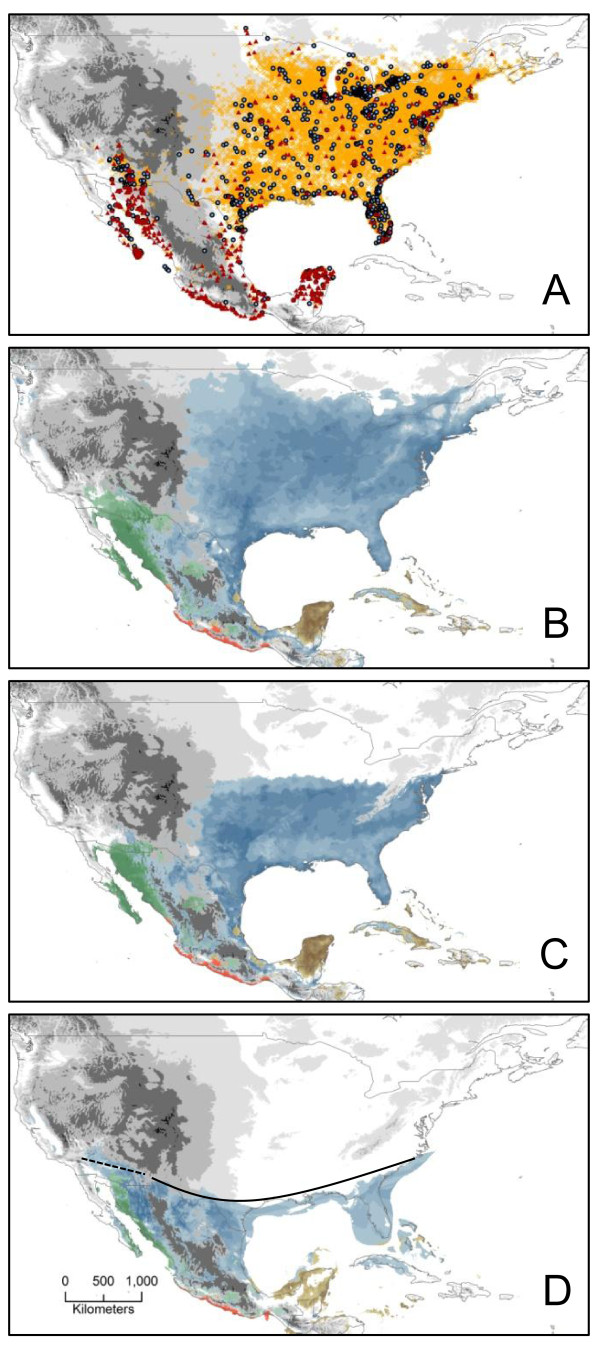
**Ecological niche models for *Cardinalis cardinalis***. A) Locality Records: field observations (Avian Knowledge Network, orange hatch marks); museum specimens (ORNIS, blue circles), records used to build ecological niche models (red triangles) B) Present-day C) Post-glacial D) Last Glacial Maximum. Dotted and solid lines show northern extent of *igenus *and *cardinalis *during the Last Glacial Maximum.

Locality records obtained from museum specimens (ORNIS) and field observations (Avian Knowledge Network) indicated that the range of *C. cardinalis *is not continuous (Figure 4A). We constructed separate models from the four clades with the following number of records; *cardinalis *(n = 232), *carneus *(n = 86), *igneus *(n = 195), and *coccineus *(n = 151). The ecological niche models produced by MAXENT performed better than random predictions and the area under the receiver operating characteristic (ROC) curve was close to one (AUC > 0.91) for the four mainland clades. Ecological niche models performed well for the two island populations based on the MAXENT diagnostics but the models did not accurately predict the small distributions of the island populations; therefore these models are not presented. The distributions generated from the present-day ecological niche models (Figure 4B) were consistent with the genetic breaks observed in our mtDNA gene tree (Figure 1). There was no suitable climatic conditions connecting *cardinalis *and *igneus *across west Texas and New Mexico, but the model predicted areas with suitable climatic conditions for *cardinalis *into the range of *igneus*. There was a predicted continuous range across the Gulf Coast connecting the *cardinalis *and *coccineus *groups, but only with the lowest suitable values projected onto the map (Figure 4B). The northern extent of the pre-19^th ^century ecological niche models for *igneus *and *cardinalis *tightly follows the northern limits of the locaily records specified in the model (Figure 4C).

The paleoecological niche model showed a pronounced reduction in the current range of the *cardinalis *clade in the eastern half of the U. S. A., which suggested that our samples in Louisiana, the Southeast, eastern México and possibly Texas are from areas that were a putative refugium (Figure 4D) during the Last Glacial Maximum. The Last Glacial Maximum model also indicated that there were suitable conditions for the *cardinalis *clade across the Chihuahuan Desert into the Sonoran Desert and east of the Sierra Madre Oriental (Figure 4D). There appears to have been a reduction in suitable climatic conditions for cardinals in México, such as on Baja California and the Mexican Pacific coast, but there was not the extreme range reduction seen in the cardinals of eastern North America.

### Spatial and Temporal Correlations

**Figure 5:**
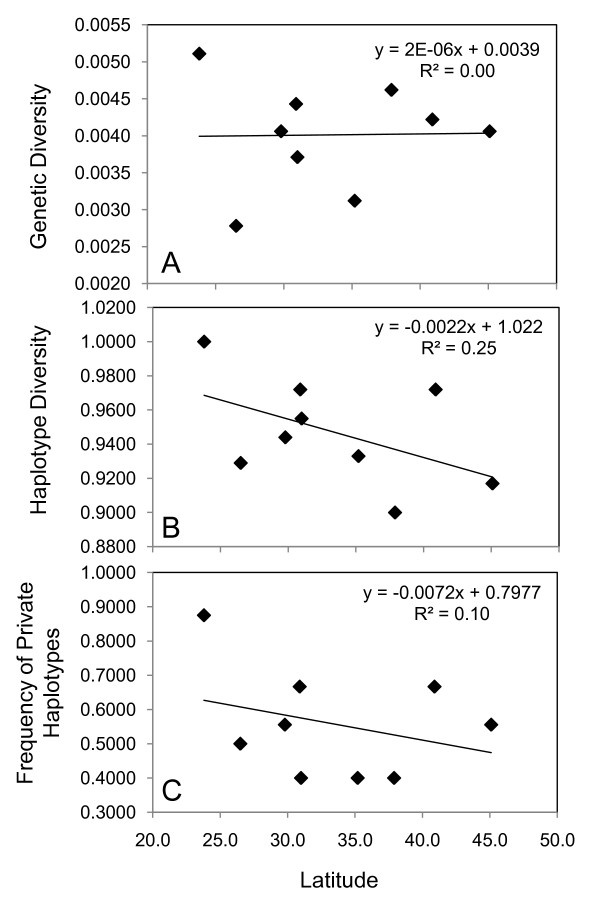
**Population-level comparison between latitude and genetic diversity metrics estimated for geographic populations**. A) Nucleotide Diversity; B) Haplotype Diversity; C) Frequency of Private Haplotypes.

**Figure 6:**
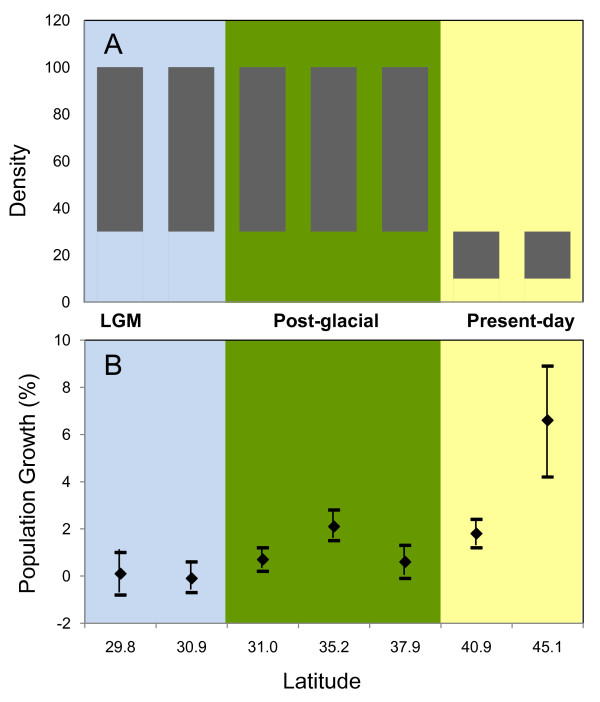
**Comparison of contemporary demographic patterns (North American Breeding Bird Survey) across the distribution of the *cardinalis *clade**. Demographic data were obtained for latitudes that had genetic samples included in the study. The colored portions represent latitudes that were inferred from the Ecological Niche Models to be stable since the Last Glacial Maximum (light blue), colonized in the Post-glacial period (green), and colonized since the 19^th ^century, Present-day (yellow). A) Population density per latitude; B) Population growth (1966-2007 with 95% CI) per latitude.

We found evidence for a positive linear relationship between clade range size and nucleotide diversity (raw values: R^2 ^= 0.83; p = 0.01; log corrected: R^2 ^= 0.51; p = 0.11; Table 1). There was no linear relationship between levels of nucleotide diversity and genetic distance (R^2 ^= 0.04; p = 0.69; Table 1). When we used nucleotide diversity estimates corrected for equal sample size, we obtained qualitatively similar results for the tests on range size and genetic distance. Within the *Cardinalis *clade neither nucleotide diversity (R^2 ^= 0.0003; p = 0.96; Figure 5A) or haplotype diversity (R^2 ^= 0.25; p = 0.17; Figure 5B) or frequency of private haplotypes (R^2 ^= 0.097; p = 0.41; Figure 5C) exhibited a linear relationship with latitude. However, there was a positive correlation with population growth and latitude (R^2 ^= 0.690; p = 0.02; Figure 6B). This pattern is also seen in contemporary population density estimates, the areas that were stable and colonized in the post-glacial period currently have a density of 30 - 100 individuals per area, while areas colonized in the 19^th ^century have 10 - 30 individuals per area (Figure 6A).

## Discussion

### Historical Signal - Phylogeographic Structure

Recent historical events, particularly Pleistocene glacial-interglacial cycles, have had a critical impact on generating phylogeographic structure in species [4]. Typically, phylogeographic studies have examined how species responded to these events within a single biome or biogeographic region [16-18]. Here, we report how a species distributed across multiple biomes and biogeographic regions of North America responded to the climatic oscillations of the Pleistocene. In the Northern Cardinal, we found six lineages that have been likely differentiating since the Pliocene. Each of the four continental clades is found in a well-established biogeographic area: the Mexican Pacific coast, the Sonoran and Peninsular deserts, eastern North America and the Yucatán Peninsula. Additionally, two insular lineages on Cozumel and the Tres Marías Islands were monophyletic.

Major geological and climatic barriers appear to define the ranges of the mainland clades. The distribution of *carneus *is constrained by the Sierra Madre del Sur to the east and habitat turnover to the north and south. *Igenus *shares a range with many co-distributed desert species that border the Sierra Madre Occidental to the east and extend to the northern and southern limits of the Sonoran Desert. This clade does not exhibit the deep genetic structure between the Sonoran and Peninsular desert populations found in other co-distributed species [19]. However, low-levels of genetic structure were detected in *igneus*, which is likely due to recent differentiation of the putative subspecies found in the Sonoran Desert, Baja California Peninsula, and Tiburón Island [20]. Present-day gene flow among cardinals in these areas is likely limited by their geographic isolation or by ecological constraints [21], but gene exchange among these groups could have been more regular during the Last Glacial Maximum when sea levels were lower [22] and populations were in closer proximity.

*Igneus *is replaced in the Chihuahuan Desert by *cardinalis *and haplotypes of both clades are found east and west of the Chihuahuan-Sonoran transition zone. This region has been identified as an area where many species exhibit genetic breaks and phylogroups often come into secondary contact [4]. Although our sampling was limited in the transition zone, we found no evidence of geographic overlap in these haplotypes and our genetic data and ecological niche model support an earlier conclusion that these forms do not hybridize [20]. The present-day ecological niche model found the geographic area between these clades unsuitable for either group, but the paleoecological niche model indicated a more continuous distribution across this gap during the Last Glacial Maximum.

The evolution of the *cardinalis *clade in eastern North America has been a history of population connectivity. Remarkably, *cardinalis*, with a range of over 3,000,000 km^2^, has no appreciable geographic structure in mtDNA, an unexpected pattern for a widely distributed clade that has several described subspecies. Comparative phylogeographic work on other widely distributed avian species has shown a similar pattern of unstructured mtDNA in eastern North American birds [23]. Alternatively, a diverse array of taxa including birds distributed in the southeast U.S.A. exhibit distinct phylogeographic structure that was shaped by a diverse array of historical factors [1].

The range limits of *cardinalis *and *coccineus *in eastern México are not well understood, but the mtDNA break coincides with the biogeographic barrier separating the Yucatán Peninsula biotic province from surrounding regions [24]. Morphological work has identified an area of turnover where *cardinalis *grades into *coccineus *in Tamaulipas [25]. Based on genetic data presented, however, specimens from Tamaulipas and areas farther south in Querétaro and Veracruz, México all possessed *cardinalis *haplotypes. Much of the Gulf Coast along México is unsuitable for cardinals because it has been converted to agriculture or encompasses low-lying areas that periodically flood. The ecological niche model also showed that habitat along the Gulf Coast to be sub-optimal. Additionally, *coccineus *appears to be a recently diverged lineage and is paraphyletic with respect to the monophyletic *saturatus*, a pattern consistent with incomplete lineage sorting between young groups [26].

The two island lineages, *mariae *and *saturatus*, are currently classified as subspecies in the *igneus *and *coccineus *groups, respectively, and both are monophyletic, suggesting that they are independently evolving lineages. Despite Cozumel being only 20 kms from the mainland and remerging 120,000 years ago [27], its biota has become distinct, with eight endemic bird and mammal species. *Mariae *occurs on the Tres Marías Islands, a small island chain 100 km off the Pacific coast of México that was likely submerged in the Late Pleistocene [28] and has several endemic subspecies [29]. Molecular dating indicated that the mean mtDNA estimates of these island lineages are "older" than the islands. The divergence of the Tres Marías Islands lineage from the mainland was not congruent with the island's geology; the 95% HPD (0.31-2.86 million years ago) covers a time period before the island was presumably under water. But, the low end of 95% HPD for the divergence of the Cozumel Island (0.07-1.06 million years ago) is consistent with geology. The incongruence between the ND2 gene tree and geology may be due to an elevated island substitution rate [30], an undetected time-dependent substitution rate [31], or coalescent variance [32]. Understanding the age of theses island lineages will require more thorough molecular dating using multilocus data.

### Mitochondrial DNA as a Phylogeographic Marker

Mitochondrial DNA is a powerful marker for identifying independently evolving populations within species because of its fast mutation rate and maternal inheritance [33]. However, incomplete lineage sorting [34], rate variation [35], and selection [36] can limit the utility of mtDNA as an evolutionary marker. Overall, the phylogeographic structure presented in this study is largely congruent with the morphological groups previously described for the species. These mtDNA lineages reflect a long history of isolation and independent evolutionary trajectories that merit further evaluation using multilocus coalescent methods [37].

Genetic diversity estimated from mtDNA is impacted by a number of factors, such as population size, age, genetic drift and mutation rate [38]. By using range size and genetic distances as proxies for population size and time of isolation, we were able to show that nucleotide diversity in *C. cardinalis *was more strongly coupled to range size than genetic distance. This relationship between range size and genetic diversity was expected because larger ranges have the potential for larger populations and thus are able to accrue higher levels of genetic diversity [39]. But, this finding is important because it suggests that mtDNA genetic diversity is at least partially coupled with cardinal population size, and indicates that the estimates provided here have not been strongly affected by selection and linkage to the W sex chromosome [40] or genetic draft [41].

### Post-glacial Expansion: Historical to Contemporary Demography

The leading edge model of post-glacial expansion predicts there will be lower genetic diversity in recently colonized regions [7]. Despite strong evidence for this model in some taxa and some geographic areas, there has not been compelling evidence for this pattern in North American birds [[17], [42], [43]; but see [44]]. These results are often attributed to other factors such as mixing of separate refugial populations, un-sampled populations, or rapid expansions. Both our genetic data and our ecological niche modeling suggest a population expansion for the *cardinalis*.

Given that the *cardinalis *clade likely expanded out of a glacial refugium, it is surprising that we found nucleotide diversity, haplotype diversity and the frequency of private haplotypes to be uniform across *cardinalis *sampling localities. Intuitively, homogeneous genetic diversity would seem attributable to birds being more vagile than other vertebrates, but this is unlikely the case here. Many birds are constrained by the same ecological barriers as non-volant organisms [45]. This ecological limitation of dispersal is evident in *igneus*, which has also undergone a recorded northern range expansion. Our *igneus *samples from Arizona/New Mexico had lower haplotype and genetic diversity than samples from Sinaloa. A critical difference between *cardinalis *and *igneus *is the amount of habitat connectivity across their ranges. There are few major dispersal barriers for cardinals in eastern North America, allowing the species to freely disperse in any direction. On the other hand, in the areas of the arid Sonoran Desert where cardinals have recently colonized, they are more restricted to habitats with suitable vegetation such as riparian zones.

The high genetic diversity in recently established populations of the eastern *cardinalis *contradicts the leading edge model and begs explanation. One suggested alternative is the Phalanx model, which posits that large populations expanded slowly into newly available habitats with no loss of genetic diversity in newly colonized areas [7]. Although the Phalanx predicts homogenous genetic diversity as seen in *cardinalis*, we see no reason for cardinals to have expanded their range slowly. Indeed, the rapid northward range expansion documented in the last 150 years suggests cardinals have high dispersal rates and can rapidly populate suitable habitat. Why may this be the case?

We suggest that cardinals in eastern North America were held at low numbers by a shortage of suitable habitat prior to the European settlement of North America. Throughout their range cardinals inhabit brushy open habitat, and prior to European settlement this ecotonal species lived in a sea of eastern hardwood forest where suitable habitat was patchy and ephemeral, created by water courses, tree falls, and fires. Throughout most of the evolutionary history of eastern cardinals, suitable habitat must have been ephemeral, leading to selection for high dispersal ability. Once European settlement converted most of Eastern North America into a vast ecotone of excellent cardinal habitat, their evolved propensity for high dispersal would homogenize genetic diversity across vast areas.

The hypothesis that cardinals exhibit high dispersal abilities is borne out by an analysis of banding records for cardinals [46]. Although conducted before root mean square dispersal was being estimated, Dow and Scott [46] found that 190 of 1523 recovered cardinals were found outside the 10-minute block of latitude and longitude where they were banded; among those that moved beyond their block, first years moved an average distance of 60 km and adults moved an average of 130 km [11]. These are large distances for a resident bird [47], and comparing them with results for North American blackbirds analyzed in modern ways [48] suggests that root mean square dispersal for Northern Cardinals is very high, possibly exceeding 100 km. Dispersal distances of this magnitude would be sufficient to homogenize genetic variation in a short period of time.

What these data suggest is that during the lag between historical expansion and the present-day, contemporary demography has played a role in shaping the observed genetic diversity pattern. Population densities are lowest at higher latitudes and population growth increases with latitude. We suggest that this population growth is not just from offspring staying in one area and increasing the density of individuals. Instead, populations are also growing as new individuals with different haplotypes are moving into new areas. Given that genetic diversity is uniform even in the youngest cardinal populations, at the highest latitudes, the lag between expansion and genetic homogenization may be on the order of decades instead of hundreds or thousands of years. As new areas became inhabitable, dispersal would likely not have been just in a northern and linear fashion, but east and west. If cardinals expanded out of southern glacial refuges rapidly and in large numbers, the signature of decreased genetic diversity may have been rapidly erased or alternatively, potentially never existed at all.

## Conclusions

In this study we used a widely distributed songbird, the Northern Cardinal, *Cardinalis cardinalis*, as a model to test historical predictions for how species evolved in response to Pleistocene glacial-interglacial cycles. We found evidence in the mitochondrial DNA and from our modeled distribution of the species that there is deep phylogeographic structure in *C. cardinalis*, which is consistent with fragmentation caused by historical climate change. This structure likely began developing well before the Late Pleistocene and is supported by the results of both contemporary and paleoecological niche models. A modeled paleodistribution along with historical demographic hypothesis tests indicated that cardinals expanded out of refugia in eastern North America since the Last Glacial Maximum. However, there is no signature of decreased genetic diversity in areas colonized after the expansion. We suggest on-going gene flow across eastern North America has likely homogenized genetic diversity across the region. These results demonstrate that both Earth history events and cotemporary processes are important in determining the geography of genetic diversity observed within species.

## Methods

We obtained samples from 163 individuals of *Cardinalis cardinalis *that includes representatives from 14 of the 18 recognized subspecies and the four subspecies groups: *carneus*, *igneus*, *coccineus*, and c*ardinalis *(Additional file 1). We included six closely related species [49] in our dataset; *C. sinuatus*, *C. phoeniceus*, *Rhodothraupis celaeno*, *Periporphyrus eyrthromelas*, *Caryothraustes poliogaster*, and *C. canadensis*) and the more distantly related *Molothrus ater *as the outgroup. We extracted total genomic DNA from tissues and toe-pads from voucher specimens using the DNeasy tissue extraction kit (Qiagen, Valencia, CA). We amplified the mitochondrial gene NADH dehydrogenase subunit II (ND2) via polymerase chain reaction (PCR) using the primers L5215 [50] and HTrpc (STRI). We used 12.5 μl reactions using the following protocol: denaturation at 94°C for 10 min, 40 cycles of 94°C for 30 s, 54°C for 45 s, and 72°C for 2 min, followed by a final 10 min elongation at 72°C. PCR products were sent to High-Throughput Genomics Unit (University of Washington, Seattle) for all subsequent sequencing steps. PCR products were purified using ExoSAP-IT (USB Corporation, Cambridge, MA), run through cycle-sequencing reactions and final products were sequenced using BigDye (Applied Biosystems, Foster City, CA) on a high-throughput capillary sequencer. We aligned chromatograms of the forward and reverse strands in Sequencher 4.9 (GeneCodes Corporation, Ann Arbor, MI) and sequences were translated into amino acids to check for premature stop codons.

### Genetic Pattern

We used several approaches to get an understanding of the distribution of genetic diversity within *C. cardinalis*. Aligned sequences were run through the program Mr. Modeltest v. 2.3 [51] to estimate an appropriate model of sequence evolution. We determined the sequence evolution model for the complete ND2 gene as well as data partitioned by codon position (1^st ^and 2^nd ^codon positions and 3^rd ^codon position). The best fit sequence model was determined by use of the Akiake Information Criterion (AIC). We constructed a gene tree using the program, MrBayes v. 3.1.2 [52]. We constructed gene trees with the non-partitioned data and partitioned by codon positions. The branch length prior was set to unconstrained with an exponential distribution with the parameter set to 100.0 to avoid artificially long branches [53]. All priors were unlinked for the partitioned data and we ran this analysis for seven million generations and sampled every 1000. We performed diagnostic tests to evaluate mixing and convergence of MCMC chains and the burn-in was determined from visual inspection of the likelihood plots in the program Tracer v 1.5 [54]. To determine which approach was the best fit to the data, we used Bayes factors, computed from the harmonic mean of the non-partitioned and partitioned posterior likelihoods. We estimated divergence times using the program BEAST v. 1. 6. 1 [55] and ran the analyses with a fixed substitution rate of 1.23 × 10^-2 ^subs/site/lineage/million years, a rate estimated relative to the avian "2% rule" [56] used for the gene cytochrome-b [see [45]]. We unlinked ND2 codon positions, and used a Yule speciation tree prior and a relaxed uncorrelated molecular clock (lognormal distribution, mean 0.0123, SD = 0.45). The analysis was run for 50 million generations and the posterior output was examined in Tracer v 1.5 [54] to assess mixing and convergence of MCMC chains.

### Genetic Diversity

Clades identified from our gene tree were used to estimate the genetic distance (internode to clade node distance) using an uncorrected p-distance for each clade in the program MEGA 4 [57]. Two of the clades, *igneus *and *cardinalis *have relatively large ranges and are comprised of several subspecies; therefore we examined the amount of genetic structure within and among subpopulations for each of these clades by performing an AMOVA (Analysis of Molecular Variance) in the program Arlequin v. 3.11 [58]. For the group designation for the AMOVA, we used U. S. states for *cardinalis *and for *igneus *we used the regions Baja California Sur, Tiburón Island, Arizona/New Mexico and Sinaloa. We calculated genetic diversity (the average number of nucleotide differences per site between two sequences), haplotype diversity [59], and the frequency of private haplotypes in the program DNAsp v. 5.10.01 [60] for each clade and for subpopulations that had adequate sample sizes (n ≥ 8). Sample sizes among clades were skewed; therefore we estimated nucleotide diversity with all available samples in a clade and with equal samples (n = 6).

### Historical Demography

To perform hypothesis tests on cardinal historical demography, we re-estimated sequence models for each individual clade that had adequate sampling (*cardinalis*, *coccineus*, and *igneus*). We evaluated demographic history in the program BEAST v. 1. 5.4 [55] by comparing the likelihood of three different models for the coalescent tree prior - constant population size, expansion growth, and Bayesian skyline. This approach allowed us to use Bayes factors to test whether a population expansion was more likely than a constant population size [61]. Given that the approach used to estimate Bayes factors in Tracer has been shown to be yield unstable results [12-14], we additionally evaluated demographic history by examining Bayesian skyline plots, which shows a visual representation of population change over time. We used the same parameter settings (except we used a strict molecular clock for this analysis) and MCMC diagnostics as our BEAST divergence time analysis. Bayes factors were calculated by manually summing the tree likelihood and coalescent/skyline columns in the BEAST log file.

### Ecological Niche Models

We downloaded locality records from two online databases, ORNIS (museum records only http://ornisnet.org) and observation records from the Avian Knowledge Network http://www.avianknowledge.net/content and plotted all records to get an estimate of the *C. cardinalis *distribution. To build an ecological niche model for *C. cardinalis*, we used only records from observations and specimens collected for this study. We assumed that these locality records are the most precise because they have been collected in the last ten years. The number of observation records for *C. cardinalis *was heavily skewed towards records in eastern North America where it is a common "backyard" bird. Therefore, we randomly sampled a subset of the records in order to have a more even sampling scheme. In total we compiled 664 records for *C. cardinalis*.

Beginning in the late 1800 s a well-documented northward range expansion has been recorded for *C. cardinalis *in the northern U. S. A. and the Colorado River Basin [11,62]. We built additional ecological niche models that attempted to approximate the pre-expansion distribution by making an estimate of their pre-19^th ^century range. The specific limits of the species' range prior to expansion are not precisely known. Therefore, based on distribution records [11], we used 40° N as a conservative and rough estimate of cardinal's pre-19^th ^century range (post-glacial) in eastern North America and 33° N in the southwest U. S. A. All records above these latitudes were not included in the analysis in the post-glacial ecological niche models.

We used the Bioclimatic variables from the WorldClim dataset (v. 1.4) with a resolution of 2.5 min [63]. Eight of the variables were correlated with other variables (R> 0.90); therefore, we used 11 of the 19 temperature and precipitation variables (BIO1, BIO2, BIO5, BIO6, BIO8, BIO9, BIO10, BIO12, BIO13, BIO15 and BIO18). We generated five replicate models for each separate clade identified from our phylogenetic analysis using the maximum entropy algorithm in MAXENT 3.3.3 [64]. The model was then applied to the Model for Interdisciplinary Research on Climate (MIROC) layers to estimate suitable climatic conditions for *C. cardinalis *during the Last Glacial Maximum. We visualized models in the program ArcGIS 9.3 (ESRI Inc., Redlands, CA). Because the distribution of *C. cardinalis *is well known we used a digital range map [65] to set the logistic threshold values for the climatic suitability for each clade and applied these same threshold values to the paleoecological niche models. To calculate range size of each clade, we converted the model raster files into polygons in ArcGIS 9.3 and recorded polygon area in square kilometers.

### Contemporary Demography

To evaluate the impact of contemporary demography on genetic diversity, we used data from the North American Breeding Bird Survey, a long-term demographic study on breeding birds in the USA and Canada [66]. Breeding Bird Survey data is based on mean counts of species along specified routes from these data density and population growth estimates are approximated. The datasets we used were population growth (% change over time) and population density estimates. We used population growth based on estimates of demographic trends of this species in a U. S. state over 40 years (1966-2007). For population densities, we directly extracted values from the Summer Distribution Map 1994-2003 using the average latitude from our genetic samples.

## Authors' contributions

All authors have read and approved final manuscript. BTS: Designed study, collected samples, generated data, performed analysis, and wrote the paper. PE: Designed study and collected samples. BEHB: Designed study and collected samples. AGNS: Designed study and collected samples. SR: Designed study, collected samples, and wrote the paper. JK: Designed study, collected samples, and wrote the paper.

## Supplementary Material

Additional file 1 **Taxon List**. List of taxa used for the study, their geographic localities, and GenBank accession numbers. Click here for file
